# Management of cryptorchidism: a survey of clinical practice in Italy

**DOI:** 10.1186/1471-2431-12-4

**Published:** 2012-01-10

**Authors:** Federico Marchetti, Jenny Bua, Gianluca Tornese, Gianni Piras, Giacomo Toffol, Luca Ronfani

**Affiliations:** 1 Institute for Maternal and Child Health IRCCS "Burlo Garofolo", Department of Paediatrics, Trieste, Italy; 2 Institute for Maternal and Child Health IRCCS "Burlo Garofolo", Department of Neonatology, Trieste, Italy; 3 Associazione Culturale Pediatri, Italy; 4 Institute for Maternal and Child Health IRCCS "Burlo Garofolo", Epidemiology and Biostatistics Unit, Trieste, Italy

## Abstract

**Background:**

An evidence-based Consensus on the treatment of undescended testis (UT) was recently published, recommending to perform orchidopexy between 6 and 12 months of age, or upon diagnosis and to avoid the use of hormones. In Italy, current practices on UT management are little known. Our aim was to describe the current management of UT in a cohort of Italian children in comparison with the Consensus guidelines. As management of retractile testis (RT) differs, RT cases were described separately.

**Methods:**

Ours is a retrospective, multicenter descriptive study. An online questionnaire was filled in by 140 Italian Family Paediatricians (FP) from *Associazione Culturale Pediatri *(ACP), a national professional association of FP. The questionnaire requested information on all children with cryptorchidism born between 1/01/2004 and 1/01/2006. Data on 169 children were obtained. Analyses were descriptive.

**Results:**

Overall 24% of children were diagnosed with RT, 76% with UT. Among the latter, cryptorchidism resolved spontaneously in 10% of cases at a mean age of 21.6 months. Overall 70% of UT cases underwent orchidopexy at a mean age of 22.8 months (SD 10.8, range 1.2-56.4), 13% of whom before 1 year. The intervention was performed by a paediatric surgeon in 90% of cases, with a success rate of 91%. Orchidopexy was the first line treatment in 82% of cases, while preceded by hormonal treatment in the remaining 18%. Hormonal treatment was used as first line therapy in 23% of UT cases with a reported success rate of 25%. Overall, 13 children did not undergo any intervention (mean age at last follow up 39.6 months). We analyzed the data from the 5 Italian Regions with the largest number of children enrolled and found a statistically significant regional difference in the use of hormonal therapy, and in the use of and age at orchidopexy.

**Conclusions:**

Our study showed an important delay in orchidopexy. A quarter of children with cryptorchidism was treated with hormonal therapy. In line with the Consensus guidelines, surgery was carried out by a paediatric surgeon in the majority of cases, with a high success rate.

## Background

Undescended testicle (UT) is present at birth with a frequency varying from 2% to 8% [1]: in Italy the estimated prevalence is 3.5% in term babies [2]. UT includes both a non-palpable testis and a palpable supra-scrotal testis which cannot be pulled down to the scrotum or does not remain there by six months of age. UT should be differed from retractile testis (RT), a condition where the testis is palpable in the supra-scrotal region but, once pulled down to the scrotum, remains there after the exhaustion of the cremasteric activity. In this case RT should not be treated. Differential diagnosis between a true RT and a supra-scrotal UT may not be easy [3].

The difficulty in achieving consensus on UT management depends largely on the long follow-up from diagnosis until the attainment of full testicular function in adulthood. An up-to-date evidence-based Consensus on the treatment of UT has been published [3] with the following recommendations: a) hormones are not recommended [4]; b) orchidopexy should be performed between 6 and 12 months of age, or upon diagnosis, if it occurs later; c) orchidopexy should be performed at centres with both paediatric surgeons or urologists and paediatric anaesthesiologists. These recommendations, in line with those by Swiss surgeons [5], are important in order to avoid adulthood consequences of UT, such as azoospermia or oligospermia [6,7], endocrine insufficiency [8,9] and possible tumoral degeneration [10-12].

Retractile testis (RT) may be more difficult to diagnose. According to the Consensus, RT should not undergo surgery but should be followed-up once yearly given the possible risk of reascent [13-15].

In Italy, current practices on UT management are little known and they appear to be heterogeneous. We hypothesised a non-adherence to the Nordic Consensus guidelines, with a delay in orchidopexy. We carried out a retrospective observational study among Italian Family Paediatricians (FP) on a cohort of children with UT in order to describe the current management of cryptorchidism in Italy, before the Consensus publication [3]. Since the differential diagnosis between UT and RT can be problematic [15], we decided to describe the management of RT cases separately.

## Methods

Ours is a descriptive and multicenter study. It enrolled FP from *Associazione Culturale Pediatri *(ACP), a national professional association represented in all Regions of Italy.

FP work independently in their offices, providing acute and chronic care to all children from 0 to 14 years old. They contract directly with the National Health System for the care of patients through a capitated reimbursement system, ensuring free paediatric care at the point of provision to all children.

ACP has been involved in previous studies and is representative of the Italian paediatric primary care system [16,17]. FP were invited to participate at a national ACP meeting, where the study protocol was first presented. Each FP was asked to fill out an electronic form for each child with UT/RT diagnosis. The form was validated by a pilot study.

The study was approved by the Independent Bioethics Committee of the Institute for Maternal and Child Health IRCCS "Burlo Garofolo" (Prot. CE/V- 86, April 28, 2008). The research was carried out in compliance with the Helsinki Declaration. Each FP asked the parents' informed consent before filling out the electronic data base.

### Inclusion criteria and data collection

We included all children born between the 1st of January 2004 and the 1st of January 2006 with both diagnosis of UT and RT. UT included both a non-palpable testis and a palpable supra-scrotal testis which could not be pulled down to the scrotum or did not remain there by six months of age. RT was defined as a palpable supra-scrotal testis which could be pulled down to the scrotum and remained there after exhausting the cremasteric activity.

At the time of our survey (early 2008), enrolled children were aged between 24 and 56 months of age. We excluded from the analysis all subjects with spontaneous resolution of UT in the first 6 months of life.

An electronic data collection form (Additional files 1 and 2) was filled in directly by each participating FP for each child with a diagnosis of cryptorchidism. Data included: age at diagnosis, clinical type according to the definitions above (non-palpable UT, palpable suprascrotal UT, RT), presence of a clinically significant concomitant disease, use of hormonal therapy, age at orchidopexy, and centre at which orchidopexy was performed.

### Outcome variables

Primary outcome variables were: mean age at orchidopexy, prevalence of hormonal therapy and of referral to a paediatric surgery centre. Secondary outcome variables were: prevalence of reported success and failure of hormonal therapy and of surgery complications.

We analyzed the data from the 5 Italian Regions with the largest number of children enrolled in order to check for any regional difference for primary outcomes.

### Data Analysis

Categorical variables are presented as absolute numbers and percentages, continuous variables as means, standard deviations, minimum and maximum range. Only means are presented as their values essentially overlapped those of medians. All data were analysed by the statistical package SPSS for Windows, version 11. Chi-square for trend was calculated to detect significant differences among regions. As a non-normal distribution of data were shown both visually and with the Kolmogorov Smirnov test, we used a non-parametric test (Mann-Whitney test or Kruskal-Wallis test in case of more than 2 independent comparisons) to compare continuous data.

## Results

One-hundred and sixty-two FP were enrolled in the study; 140 (86%) responded to the questionnaire. Mean age of enrolled FP was 51,7 years (SD 4,7), with a mean of 26.3 years of clinical experience (SD 5.1) after medical degree. A mean of 895 (SD 148) patients were followed by each FP. Participating FP were representative of the whole country as they came from 18 out of the 20 Italian administrative regions.

**Figure 1:**
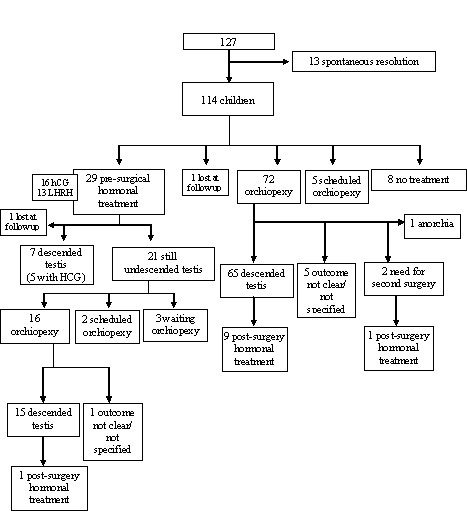
**Data flow diagram describing children with a diagnosis of UT**.

Eighty-three out of 140 FP (59%) followed at least 1 patient with UT or RT in the defined period, adding up to a total of 177 children. After the exclusion of 3 cases who did not meet the inclusion criteria and 5 cases with spontaneous resolution of cryptorchidism in the first 6 months of age, 169 children were considered in the final analysis: 127 with UT and 41 with RT (1 with unknown position). Figure 1 and Table 1 show the main results of the study.

**Table 1:** Main outcome measures for children with cryptorchidism as a whole, and divided in UT and RT subgroups.

	UT #	RT #
**Spontaneous resolution**	**13/127 (10%)**	**14/41 (34%)**
- Mean age at resolution, in months (SD; range)	21.6 (10.8; 9.6-40.8)	27.6 (13.2; 6-46.8)
- Age at spontaneous resolution, categories:		
• Between 6 and 11 months	4 (31%)	2 (14%)
• Between 12 and 23 months	3 (23%)	5 (36%)
• 24 months or more	6 (46%)	7 (50%)

**Hormonal treatment (as pre-surgical or unique treatment)**	**29/127 (23%)**	**4/41 (10%)**
- hCG	16/29 (55%)	2/4 (50%)
- LHRH	13/29 (45%)	2/4 (50%)
**Mean age at beginning of hormonal treatment, in months **(md = 1) (SD; range)	20.4 (13.2; 6-50.4)	30 (7.2; 20.4-36)
- Mean age at beginning of hCG, in months (md = 1)	24 (13.2; 6-50.4)	30 (3.6; 43.2-33.6)
- Mean age at beginning of LHRH, in months	15.6 (10.8.; 6-42)	28.8 (10.8; 20.4-36)
**Descend of testis after hormonal treatment**	7/28 (25%)*	**1/4 (25%)**

**Surgical treatment**	**88/126 (70%)**	10/40 (25%)**
- Mean age at surgery, in months (md = 1) (SD; range)	22.8 (10.8; 1.2-56.4)	27.6 (10.8; 12-46.8)
- Age at surgery, categories (md = 1):		
• before 6 months	3 (3%)	**-**
• between 6 and 11 months	9 (10%)	1 (10%)
• between 12 and 23 months	44 (51%)	4 (40%)
• 24 months or more	31 (36%)	5 (50%)
- Carried out in paediatric surgery center	79/88 (90%)	8/10 (80%)
- Carried out in paediatric surgery center < 12 months	12/12 (100%)	1/1 (100%)
- Carried out in the Region where child lived (md = 4)	74/85 (87%)	8/9 (89%)
- Scheduled orchidopexy	7/126 (6%)	4/40 (10%)
**Surgery outcome**		
- Descended testis	80/88 (91%)	10/10 (100%)
- Need for second surgery	2/88 (2%)	-
- Anorchia and prosthesis positioning	1/88 (1%)	-
- Outcome not clear/not specified	5/88 (6%)	-

**Post-surgery hormonal treatment**	**11/126 (9%)**	**-**
- hCG	10/11 (91%)	
- LHRH	1/11 (9%)	

Categorical variables are presented as percentages, continuous variables as mean (SD; range).

* 1 child lost at follow up from FP after beginning of treatment

** 1 child lost at follow up after sending to surgeon

§ 17 children after hormonal treatment; 82 underwent surgery as first line treatment

# 1 child without indication of physical examination and clinical type

### Characteristics of children with UT

Mean birthweight was 3262 grams (SD 615) and gestational age 39 weeks (SD 2.1), with the majority of cases (88%) born between 37 and 41 weeks.

Sixteen percent of children had bilateral cryptorchidism, while 45% had right and 33% left cryptorchidism. Seventy-two (57%) were in inguinal position, while 55 (43%) were non-palpable. In 18% of cases (n = 23) there was an associated disease or syndrome. The mean age at last follow-up was 35.1 months (SD 13.1, range 4.8-58.8; 5 missing data).

### Hormonal therapy

Overall 39 out of 127 children (31%) were treated with hormones, of whom 29 before surgery and 10 after surgery (1 both pre- and post-surgery), although use of hormones was not homogenous among regions (χ^2 ^for trend, p = 0,001) (Table 2).

**Table 2:** Regional differences in the use of hormonal therapy, use of surgery and mean age at surgery

	Hormonal therapy*	Surgery**	**Mean age at surgery**, **in months (SD)**†
*Friuli-Venezia Giulia*	18.2% (2/11)	90% (9/10)§	21,5 (± 8,0)

*Veneto*	9.4% (3/32)	71.9% (23/32)	23,9 (± 10,85)

*Lombardy*	30% (6/20)	75% (15/20)	21,2 (± 10,6)

*Umbria*	41.7% (5/12)	58.3% (7/12)	23,6 (± 18,7)

*Sicily*	53.3% (8/15)	46.7% (7/15)	25,0 (± 6,6)

* χ^2 ^per trend, p = 0,001; ** χ^2 ^per trend, p = 0,026; † p = 0.7, §1 children lost at follow up after starting hormonal therapy

The mean age of initiation of pre-surgical hormonal therapy was 20.4 months. Of 29 cases who underwent hormonal treatment before surgery, 16 were treated with human chorionic gonadotropin (hCG), 13 with luteinizing hormone-releasing hormone (LHRH). No child was treated with both drugs. Hormonal treatment led to resolution in 7/28 cases (25%), of whom 5/15 (33%) treated with hCG and 2/13 (15%) treated with LHRH; 1 was lost at follow up.

### Orchidopexy

Overall 88 out of 126 children with UT (70%, missing data = 1) underwent orchidopexy, 72 without previous hormonal treatment, 16 with. The use of surgery varied through regions (χ^2 ^for trend, p = 0,026) (Table 2) and was not related to the age of FP (p = 0.3).

Mean age at intervention was 22.8 months. Mean age of intervention did not differ between was those who underwent surgery without receiving a previous hormonal treatment (mean of 21.6 months, SD 12, range 1,2-56,4 months) and those who received hormones before surgery (mean of 24 months, SD 8.4, range 8.4-37.2) (p = 0.2).

Overall only 13% of children underwent surgery within the first year of life, while 36% at more than 2 years of age.

In 80 out of 88 cases (91%), surgery resulted in the resolution of cryptorchidism (Figure 1). Only 2/16 children (12.5%) underwent surgery within the first year of life. No surgical complications were reported.

### Paediatric surgery centre

In 90% of cases, orchidopexy was carried out by a paediatric surgeon, in 100% of cases in children younger than 12 months. The 2 cases who needed a second surgery came both from a paediatric surgery centre. In 87% of cases, children underwent orchidopexy in the same region from where they came.

### Characteristics of children with RT

Among 41 children with diagnosis of RT, 14 had a spontaneous resolution at a mean age of 27.6 months. Four children were treated with hormones which was started at a mean age of 30 months with a success rate of 25%. Ten children with RT underwent orchidopexy at a mean age of 27.6 months. In 9 cases surgery was conducted as first line treatment, in 1 it was carried out in a paediatric surgery center, in 1 before the age of 12 months, with an overall success rate of 100%. Four children had scheduled surgery, 10 are followed up without any treatment, 1 child was lost at follow-up (Table 1).

## Discussion

Cryptorchidism is a relevant condition, with an estimated incidence in Italy of 3,5% yearly [2]. For unknown reasons, UT prevalence appears to have increased in some countries [18,19]. This increase is of concern given the long term adverse health effects of UT, such as altered semen quality [4,20], endocrine insufficiency [8,21,22] and increased risk of testicular cancer [11,23-27]. In fact cryptorchidism has been proposed to be part of a "testicular dysgenesis syndrome" which includes hypospadias, reduced semen quality and testicular cancer. These conditions are thought to have a common origin in prenatal testicular maldevelopment, which affects both Leydig and Sertoli cells and germ cell differentiation [28]. The aim of an early orchidopexy is to prevent the possible adulthood consequences on spermatogenesis [3,29,30], while its effect on the risk of testicular cancer remains to be established.

Our study aim was to describe the clinical practice of cryptorchidism management in Italy before the publication of the Consensus [3] by selecting a cohort of children born and treated before then, with the additional aim of divulgating the Consensus. In order to reflect the Italian National Health System, we chose to draw our study sample from the general population followed at the primary care level from FP, retrieving a total of 169 cases, of which 127 with UT and 41 with RT.

Our study showed that one out of 4 children with UT have been treated with hormones (55% of patients treated with hCG, 45% with LHRH) as first line therapy at a mean age of 21,6 months. Success rate of both hormones was 25%, in line with those published in the literature [31]. According to the Nordic Consensus Guidelines hormones are not formally recommended [3,4]. Reported success rate for hormones is of 15-20% compared to 95% for surgical treatment [3,4,31]. The Nordic Consensus reservations over hormones use relate to the fact that hCG has been associated not only with an increased risk of testicular damage and consequent reduced spermatogenesis [31-35] attributed to an increased germ cells apoptosis, but also with systemic effects, such as ventricular hypertrophy [36].

However, the aspect of hormonal use is still debated, and the *European Society of Paediatric Urologists' *(ESPU) is in favour of using gonadotrophin releasing hormone (GnRH) analogues to improve fertility in boys UT, particularly in those with bilateral disorders [37]. Although the number of studies is limited and patient numbers are relatively low, GnRH analogues (in some studies used in combination with hCG) do appear to have a statistically significant beneficial effect on fertility indices both when used before orchidopexy [38,39] and after [40,41]. Randomised controlled trials are warranted to confirm the beneficial effect of the adjuvant GnRh therapy [42].

We found a delay with regard to the recommended timing of orchidopexy, as first line treatment (mean age at surgery of 21.6 months) and even longer as secondary treatment after hormones (mean age at surgery of 24 months), although the difference was not significant. Only 13% of UT cases underwent orchidopexy before one year of age; in 1 out of 3 cases surgery was performed after 2 years. The delay in the surgical approach of UT is a common worldwide practice. An Italian survey conducted in 2001 showed a mean age at orchidopexy of 27,8 months, with 16% of not yet treated children by the age of 32 months [43]. In UK between 1997 and 2005 the percentage of children with UT undergoing surgery before 2 years of age increased slightly from 15,8% to 28.5% [44]. In USA, a recent survey showed a rate of age at orchidopexy similar to our study: 18% by 1 year of age, and 43% by the age of 2 [45]. This latter study found a significant association of race and insurance payer with age at orchidopexy. In Italy the delay of surgery cannot be imputable to insurance status, as all citizens have a public insurance or to race, given that around 93% of Italian citizens are Caucasian. This suggests that in Italy the reasons for the delay in orchidopexy rather reflect differences in the knowledge and updating of the clinicians, as well as organizational aspects at the national and regional level. With this respect, we found significant differences in the management of UT among Italian regions.

In line with the Consensus recommendations we found a very high percentage of referral to a paediatric surgeon: 9 out of 10 UT cases were treated in a tertiary centre by a paediatric surgical team (100% under the first year of life) with a high rate of success (91%), similarly to that reported by others [46]. Orchidopexy was described as a safe surgical treatment as no complications were reported.

Our study also described 41 children with an initial supposed diagnosis of RT. It showed a high percentage of resolution of RT cases during follow-up, as it occurs in RT natural history. Nevertheless, surprisingly 10 children with initial diagnosis of RT underwent surgery and 4 were treated with hormones. This may be explained by two main reasons. Firstly, patients with significant testicular retractility appear to be at a high risk for acquired cryptorchidism [13]: for this reason according to the Consensus an annual follow-up of RT cases throughout childhood is recommended until puberty due to the high risk of reascent [3,14,44]. Secondly, the initial diagnosis, which distinguishes between a true condition of UT from RT, still represents a diagnostic dilemma, and the problem of misclassification is well known [15] requiring specific training. The data that 13 children with a reported diagnosis of UT had a spontaneous resolution seems to confirm this difficulty in the UT-RT differential diagnosis.

Other than the possible diagnostic problems mentioned above, our study is not without limitations. It is a retrospective survey, the nature of which cannot rule out a recall bias of the collected data. However, all FP involved could retrieve data for all patients from an electronical clinical file. Adhesion to the study was on a voluntary basis representing a possible bias as our results may not be representative of the management of cryptorchidism in the whole Italian reality. However, there was a high response rate from the FP invited to participate to the study (86%), and the participating FP had a countrywide distribution. Differences according to Italian regions were noted highlighting that current practices on cryptorchidism management are not homogenous and that an update on cryptorchidism management is auspicable on a national basis.

## Conclusions

The correct and timely management of children with cryptorchidism is important to promote gonadal development and to avoid the adulthood consequences of spermatogenesis defects. The results of our study allowed for the first time to describe how was the management of cryptorchidism in Italy before the publication of the Consensus. Hormone therapy was used in about one quarter of the cases. The average age at surgery was much higher than recommended in the Consensus, and only 13% of children underwent surgery before 12 months of age.

Our results suggest that the spread of the Consensus guidelines on the management of cryptorchidism among all paediatricians is warranted in order to reduce age at orchidopexy and to have a more homogenous approach in the whole of Italy.

## Abbreviations

UT: undescended testis; RT: retractile testis; FP: family paediatrician; hCG: human chorionic gonadotropin; LHRH: luteinizing hormone-releasing hormone.

## Competing interests

The authors declare that they have no competing interests.

## Authors' contributions

FM, JB and G. Tornese conceived the study, participated to its design and coordination, and wrote the first draft of the manuscript. LR participated to the design of the study, its coordination and performed the statistical analysis. GP designed the online questionnaire and carried out the data collection. G. Toffol participated to the study design and promoted the study among ACP. All authors read and approved the final manuscript.

## Pre-publication history

The pre-publication history for this paper can be accessed here:

http://www.biomedcentral.com/1471-2431/12/4/prepub

## Supplementary Material

Additional file 1 **Italian data collection form**. Data collection form in Italian language as in the original electronic form. Click here for file

Additional file 2 **English data collection form**. Data collection form translated in English. Click here for file
