# Supplementary material for: Management of cryptorchidism: a survey of clinical practice in Italy
Source: BMC Pediatr. 2012 Jan 10;12:4. doi: 10.1186/1471-2431-12-4 (PMC3295675; doi:10.1186/1471-2431-12-4)
Supplement: Additional file 1 — Italian data collection form. Data collection form in Italian language as in the original electronic form. [file 1471-2431-12-4-S1.DOC]

**Additional file 1 – Data collection form in Italian language as in the original electronic form**

***Scheda Pediatra***

Nome:

Cognome:

Età:

Anno di Laurea:

Luogo di Lavoro:

Regione:

Numero attuale di assistiti:

Numero di bambini maschi nati fra il 1° gennaio 2004 e il 1° gennaio 2006:

Fra questi, indicare numero di bambini con testicolo ritenuto:

***Scheda da compilare per ogni bambino con testicolo ritenuto***

Data di nascita (gg/mm/aaaa)

Età gestazionale (settimane + giorni)

Peso alla nascita (in grammi)

Criptorchidismo alla nascita: sì-no-non so

Madre fumatrice in gravidanza: sì-no-non so

Diabete gestazionale: sì-no-non so

Sindromi correlate: sì-no-non so

- se sì, specificare quale:

Altre anomalie associate: sì-no-non so

- se sì, specificare quale:

Data diagnosi criptorchidismo (gg/mm/aaaa)

Criptorchidismo: destro – sinistro – bilaterale

All’esame obiettivo al momento della diagnosi: Testicolo soprascrotale (non palpabile o inguinale) - Testicolo retrattile

Data ultimo follow up (gg/mm/aaaa)

Trattamento:

- risoluzione spontanea: sì – no

- Se sì, data della risoluzione (gg/mm/aaaa)

- terapia ormonale: sì – no

- se sì:

- hCG (dosaggio - n° somministrazioni)

- LHRH (dosaggio - n° somministrazioni)

data inizio trattamento (gg/mm/aaaa)

data fine trattamento (gg/mm/aaaa)

esito del trattamento: disceso - non disceso

se non disceso: attesa – chirurgia

- terapia chirurgica: sì - no - in programma

- se sì:

data intervento (gg/mm/aaaa)

intervento effettuato presso una chirurgia pediatrica? sì – no; della regione - fuori regione

Note
