# Supplementary material for: Management of cryptorchidism: a survey of clinical practice in Italy
Source: BMC Pediatr. 2012 Jan 10;12:4. doi: 10.1186/1471-2431-12-4 (PMC3295675; doi:10.1186/1471-2431-12-4)
Supplement: Additional file 2 — English data collection form. Data collection form translated in English. [file 1471-2431-12-4-S2.DOC]

**Additional file 2 – Data collection form translated in English**

***Family paediatrician form***

First Name:

Surname:

Age:

Year of degree:

Place of Work:

Region:

Current number of patients followed:

Number of male children born between 1^st^ January 2004 and 1^st^ January 2006:

Among these, indicate number of children with undescended testis:

***Form to be completed for each child with undescended testis***

Date of birth (dd/mm/yyyy)

Gestational age (weeks + days)

Birth weight (in grams)

Cryptorchidism at birth: yes – no – don’t know

Did mother smoke during pregnancy? yes – no – don’t know

Gestational diabetes: yes – no – don’t know

Correlated syndromes: yes – no – don’t know

- if yes, please specify:

Other associated anomalies: yes – no – don’t know

- if yes, please specify

Date of cryptorchidism diagnosis (dd/mm/yyyy)

Cryptorchidism: right – left – bilateral

On physical examination at the time of diagnosis: Supra-scrotal testis (non-palpable or inguinal) - Retractile testis

Date at last follow-up (dd/mm/yyyy)

Treatment:

- spontaneous resolution: yes – no

- if yes, date of resolution: (dd/mm/yyyy)

- hormonal treatment: yes – no

- if yes,

- hCG (dosage - no. of doses)

- LHRH (dosage - no. of doses)

treatment start date (dd/mm/yyyy)

treatment end date (dd/mm/yyyy)

treatment outcome: descended – undescended

if undescended: wait-and-see – surgery

- surgical treatment: yes - no - scheduled

- if yes, surgery date: (dd/mm/yyyy)

- was orchiopexy conducted in a paediatric surgery? yes – no; in your region – in another region

Notes
